# High Incidence and Duration of Antibiotic Use Among a Cohort of Men Who Have Sex With Men in Seattle, Washington

**DOI:** 10.1093/ofid/ofaf051

**Published:** 2025-01-30

**Authors:** Gregory K Zane, Lindley A Barbee, Ann Duerr, Matthew R Golden, Lisa E Manhart, Dobromir Dimitrov, Christine Khosropour

**Affiliations:** Department of Epidemiology, University of Washington, Seattle, Washington, USA; Department of Medicine, University of Washington, Seattle, Washington, USA; HIV/STD Program, Public Health–Seattle and King County, Seattle, Washington, USA; Department of Epidemiology, University of Washington, Seattle, Washington, USA; Department of Global Health, University of Washington, Seattle, Washington, USA; Vaccine and Infectious Disease Division, Fred Hutchinson Cancer Center, Seattle, Washington, USA; Department of Epidemiology, University of Washington, Seattle, Washington, USA; Department of Medicine, University of Washington, Seattle, Washington, USA; HIV/STD Program, Public Health–Seattle and King County, Seattle, Washington, USA; Department of Epidemiology, University of Washington, Seattle, Washington, USA; Department of Global Health, University of Washington, Seattle, Washington, USA; Vaccine and Infectious Disease Division, Fred Hutchinson Cancer Center, Seattle, Washington, USA; Department of Applied Mathematics, University of Washington, Seattle, Washington, USA; Department of Epidemiology, University of Washington, Seattle, Washington, USA

**Keywords:** antibiotics, cohort study, doxycycline postexposure prophylaxis, epidemiology, men who have sex with men

## Abstract

**Background:**

Doxycycline postexposure prophylaxis (doxy-PEP) effectively prevents bacterial sexually transmitted infections (STIs) but may increase antibiotic pressure. Little is known about longitudinal antibiotic use among men who have sex with men (MSM), a key population for doxy-PEP.

**Methods:**

We analyzed data from a prospective cohort of MSM in Seattle, Washington, from 2016 to 2018, prior to the introduction of doxy-PEP. Antibiotic use and reason for prescription were self-reported in weekly surveys and extracted from medical records. We characterized antibiotic use across 49 weeks of follow-up, stratified by specific antibiotics of interest and reasons for prescription. Incidence rates (IRs) were calculated for the number of incident events of antibiotic initiation per 100 person-years (PY) at risk. We assessed factors associated with antibiotic initiation using negative binomial regression to estimate adjusted incidence rate ratios (IRRs).

**Results:**

Among 140 participants, 68.6% (n = 96) received at least 1 antibiotic during follow-up, resulting in an overall IR of 264.5 events of antibiotic initiation per 100 PY and 1696 total days of antibiotic use. STI treatment was the most common reason for antibiotic initiation (IR, 153.5 events per 100 PY; 462 days); however, treatment for other conditions contributed most to overall days of antibiotic use (IR, 42.6 events per 100 PY; 947 days). An age of 25–39 years (IRR, 1.54 [95% confidence interval {CI}, 1.02–2.32]) and a history of bacterial STIs <12 months prior to enrollment (IRR, 1.81 [95% CI, 1.12–2.93]) were significantly associated with higher incidence of antibiotic initiation.

**Conclusions:**

Antibiotic consumption among this population was very high. Our analysis provides a necessary foundation for assessing the potential impacts of doxy-PEP.

## Teaser

In this Seattle-based cohort of men who have sex with men, nearly 70% of individuals received at least 1 antibiotic during 49 weeks of follow-up, resulting in an overall incidence of 264.5 events of antibiotic initiation per 100 person-years.

Sexually transmitted infections (STIs), including *Chlamydia trachomatis* (CT), *Neisseria gonorrhoeae* (GC), and syphilis, persist as significant contributors to global morbidity among individuals of reproductive age [1, 2]. In recent years, the incidence of STIs has increased substantially among gay, bisexual, and other men who have sex with men (MSM) [1, 3]. This change is likely a result of several factors, including increased uptake of human immunodeficiency virus (HIV) preexposure prophylaxis (PrEP), associated increases in STI testing frequency, and reductions in condom use [4–6].

In response to rising STI rates, doxycycline postexposure prophylaxis (doxy-PEP) has emerged as a new biomedical STI prevention strategy involving the use of 200 mg of doxycycline within 72 hours after sex acts [7]. Doxy-PEP is effective among MSM in preventing syphilis, CT, and to a lesser extent GC, with reductions in the composite incidence of bacterial STIs ranging from 47% to 82% [8–10]. Organizations, including the United States Centers for Disease Control and Prevention (CDC), have endorsed doxy-PEP as an STI prevention strategy among eligible MSM and transgender women [7, 11, 12].

However, there are potential drawbacks to widespread doxy-PEP use, including the potential for the development of antibiotic resistance in STIs and nontarget organisms [13–15]. Further, the introduction of doxy-PEP may increase or decrease the cumulative use of antibiotics in MSM. At present, antibiotic use patterns among MSM have been underexamined, with available data being cross-sectional [16]. Establishing an understanding of longitudinal antibiotic use behaviors will allow us to assess how doxy-PEP and changing standards of STI screening and treatment affect antibiotic consumption and risk of resistance dissemination in this population.

Among a cohort of MSM in Seattle, Washington, observed prior to the popularization of doxy-PEP, we aimed to determine the incidence and correlates of antibiotic initiation, and duration of antibiotic use.

## METHODS

### Study Design and Population

We analyzed antibiotic use data collected through the ExGen Study, a 48-week prospective, natural history study of extragenital GC/CT conducted between March 2016 and December 2018 [17]. MSM from the Public Health–Seattle and King County Sexual Health Clinic (PHSKC) and the University of Washington's Center for AIDS Research patient registry were eligible and recruited if they were at least 18 years of age, English-speaking, had access to the internet, reported receptive anal sex in the last 12 months, performed oral sex in the past 2 months, and met at least 1 of the following criteria: (1) had a diagnosis of GC, CT, or syphilis in the past 12 months; (2) reported using methamphetamine or amyl nitrate in the past 12 months; or (3) reported having at least 2 sexual partners in the past 2 months or at least 5 sexual partners in the past 12 months. All enrolled study participants provided written informed consent for participation and medical record extraction. This study was approved by the University of Washington's Institutional Review Board (number 50028).

### Study Procedures

ExGen Study procedures have been previously described [17–19]. In brief, participants attended an in-person enrollment visit where demographic, clinical, and sexual behavior data were collected. Participants were then tested for STIs and HIV; those who tested positive for an STI at enrollment were treated.

During the 48-week follow-up period, each week participants completed a personalized, password-protected electronic survey on the REDCap platform [20, 21]. Weekly survey questions asked participants about hygiene practices, sexual behaviors, symptoms, STI testing, medication, and antibiotic use since their last survey. Participants also self-collected weekly pharyngeal and rectal specimens, which were tested for GC/CT after participants completed the study. After completion of follow-up, study personnel extracted data on STI diagnoses and treatments from participants’ medical records through PHSKC and the Madison Clinic, an HIV primary care clinic where many of the participants received care. At the final in-person visit, participants completed a final survey and underwent STI testing and treatment. Participants were compensated for completing weekly surveys and shipping self-collected specimens.

### Measures of Antibiotic Use

We collected information on antibiotic use in 2 ways. First, participants were asked in weekly surveys to self-report their antibiotic use in the past week, including the name, route of administration, and reason for use. Second, study personnel collected information on antibiotic use during the duration of the study from participants’ medical records, and recorded the type of antibiotic, treatment modality, and clinical indication.

### Definitions and Statistical Analyses

#### Incidence of Antibiotic Initiation

We defined an incident event as the initiation of an antibiotic regimen at enrollment or participant-reported initiation of antibiotics at any point during follow-up, regardless of the length of treatment course. Receipt of antibiotics during follow-up not reported by study participants during weekly surveys but identified in the medical record was also included as an incident event. If 2 or more antibiotics were initiated at the same time, each antibiotic was counted as a separate initiating event. We further stratified initiation by antibiotic type (doxycycline, azithromycin, ceftriaxone, or benzathine-penicillin) and reason for antibiotic use (STI diagnosis, epidemiologic treatment for STI contact, or other reported health conditions). Although additional antibiotics were reported, the 4 antibiotics above were selected due to their frequency of use in the cohort (∼87.3% of all antibiotics initiated) and their importance in STI treatment. In secondary analyses, we stratified antibiotic initiation by age (<25, 25–39, ≥40 years), HIV status and PrEP use at enrollment, and reported STI diagnosis within 12 months prior to enrollment. Study participants were assigned 49 weeks of person-time at risk (1 week preenrollment; 48 weeks postenrollment), as antibiotic initiation could be evaluated through medical records for weeks when individuals did not complete a weekly survey or were lost to follow-up. For stratification by antibiotic type, the time when individuals were receiving the specific antibiotic under consideration was excluded from the denominator (Supplementary Table 1). We also excluded a clinically relevant, antibiotic-specific period postregimen completion where a study participant would likely not be prescribed the antibiotic again. Incidence rates (IRs) were calculated as the number of incident events per 100 person-years (PY) at risk. Exact rate ratio tests evaluated differences in unadjusted IRs across strata.

#### Duration of Antibiotic Use

We estimated the duration of antibiotic use as the number of days where antibiotics were prescribed for (1) treatment at enrollment, (2) self-reported use of antibiotics at any point during follow-up, or (3) unreported use of antibiotics at any point during follow-up extracted from medical records. If 2 or more antibiotics were initiated at the same time, the duration of each antibiotic was counted separately. We stratified days of antibiotic use by antibiotic type and reason for use, as well as by age, HIV status and PrEP use at enrollment, and reported STI diagnosis within 12 months before enrollment. We estimated lower- and upper-bound estimates to account for established ranges in antibiotic course duration (Supplementary Table 1). To account for person-time at risk, we estimated IRs for days of antibiotic use per 100 PY at risk. Study participants each contributed 49 weeks of person-time at risk. However, to estimate IRs stratified by antibiotic type, time was excluded when individuals were either receiving the specific antibiotic under consideration or were within the clinically relevant time postregimen where they would not be prescribed the antibiotic again (Supplementary Table 1).

#### Factors Associated With Antibiotic Initiation

To explore the factors associated with antibiotic initiation, we used negative binomial regression analysis with generalized estimating equations with an exchangeable correlation structure to account for within-cluster correlation among observations. In primary analyses, we used overall antibiotic initiation, defined previously, as the primary outcome of interest. We selected the following a priori predictor variables: age (<25, 25–39, ≥40 years), race/ethnicity, HIV status and PrEP use at enrollment, history of chronic medical conditions, reported methamphetamine use, and STI diagnosis within 12 months of enrollment. To ensure model convergence, we collapsed individuals who reported race/ethnicity as “non-Hispanic Asian/Pacific Islander,” “another race/ethnicity,” or “unknown” into a single category of “another race/ethnicity.” Any variables with *P* < .2 in univariate analyses were included in the multivariate model. Results of univariate and multivariate regressions are presented as incidence rate ratios (IRRs). In secondary analysis, factors associated with antibiotic initiation for STI treatment alone were explored using the same approach. An α ≤.05 was considered statistically significant for multivariate analyses. Statistical analyses were performed in R version 4.1.1 software.

## RESULTS

Between March 2016 and December 2018, 140 MSM were enrolled into ExGen, collectively contributing 131.6 PY of follow-up. The median age was 35 years, 63% reported non-Hispanic White race, and 51% were people living with HIV (Table 1). Among participants living without HIV, 58% reported using HIV PrEP.

**Table 1.** Baseline Demographic and Clinical Characteristics of Study Participants in the ExGen Study, 2016–2018 (N = 140)

Characteristics	Study Participants (n = 140)	Study Participants With ≥1 Antibiotic Regimen Initiation (n = 96)
Age, y, median (IQR)	35 (28–46)	33 (28.0–44)
Race/ethnicity		
Non-Hispanic White	88 (62.9)	62 (64.6)
Non-Hispanic Black/African American	15 (10.7)	9 (9.4)
Non-Hispanic Asian/Pacific Islander	8 (5.7)	6 (6.2)
Hispanic/Latinx	25 (17.9)	18 (18.8)
Another race/ethnicity	3 (2.1)	0 (0.0)
Unknown	1 (0.7)	1 (1.0)
Income		
≤$14 999	54 (38.6)	32 (33.3)
$15 000–$29 999	26 (18.6)	18 (18.8)
$30 000–$49 999	28 (20.0)	22 (22.9)
$50 000–$99 999	25 (17.9)	19 (19.8)
≥$100 000	7 (5.0)	5 (5.2)
Educational attainment		
Grade school	3 (2.1)	2 (2.1)
High school	30 (21.4)	21 (21.9)
Some college/associate or technical degree	50 (35.7)	32 (33.3)
Bachelor's degree/some graduate school	39 (27.9)	27 (28.1)
Graduate degree	18 (12.9)	14 (14.6)
HIV status and PrEP use		
Living with HIV	71 (50.7)	49 (51.0)
Living without HIV, on PrEP	40 (28.6)	30 (31.3)
Living without HIV, not on PrEP	29 (20.7)	17 (17.7)
Reported chronic medical condition	86 (61.4)	58 (60.4)
Criteria for study entry		
Bacterial STI diagnosis, <12 mo	104 (74.3)	77 (80.2)
Methamphetamine or popper use, <12 mo	35 (25)	21 (21.9)
No. of sexual partners^a^	117 (83.6)	86 (89.6)

Data are presented as No. (%) unless otherwise indicated.

Abbreviations: HIV, human immunodeficiency virus; IQR, interquartile range; PrEP, preexposure prophylaxis; STI, sexually transmitted infection.

^a^Reported as at least 2 sexual partners in the past 2 months or at least 5 sexual partners in the past 12 months.

### Incidence of Antibiotic Initiation

**Figure 1.**
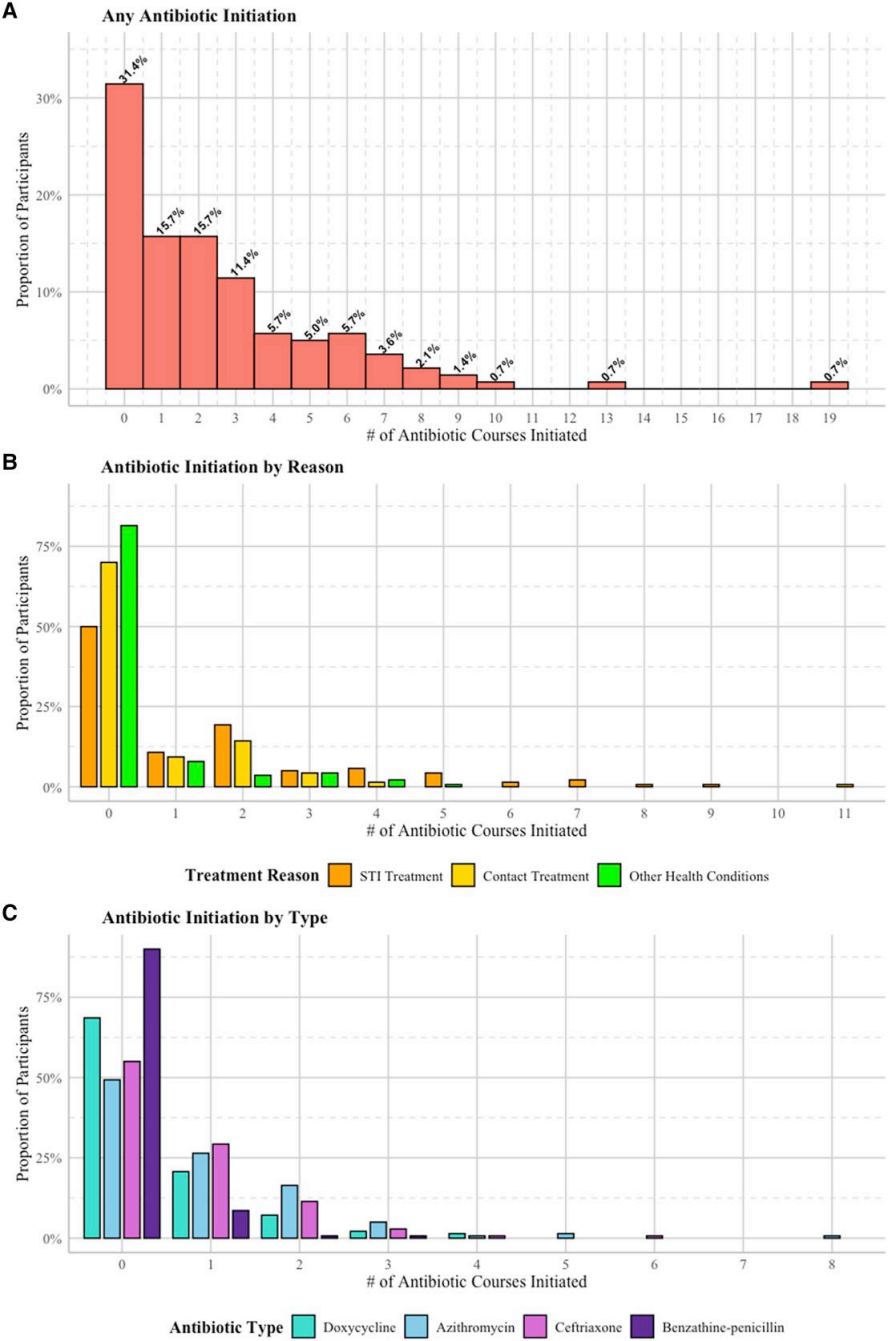
Distribution of antibiotic initiation events during follow-up in the ExGen Study, stratified by any antibiotic initiation, treatment reason, or antibiotic type. Abbreviation: STI, sexually transmitted infection.

During follow-up, 69% (n = 96) of participants received at least 1 antibiotic, with 16% initiating 1 regimen, 33% initiating 2–4, and 20% initiating 5 or more (Figure 1). Of all participants, 50%, 30%, and 19% reported at least 1 antibiotic for the treatment of STIs, presumptive treatment as an STI contact, or for other health conditions, respectively.

**Figure 2.**
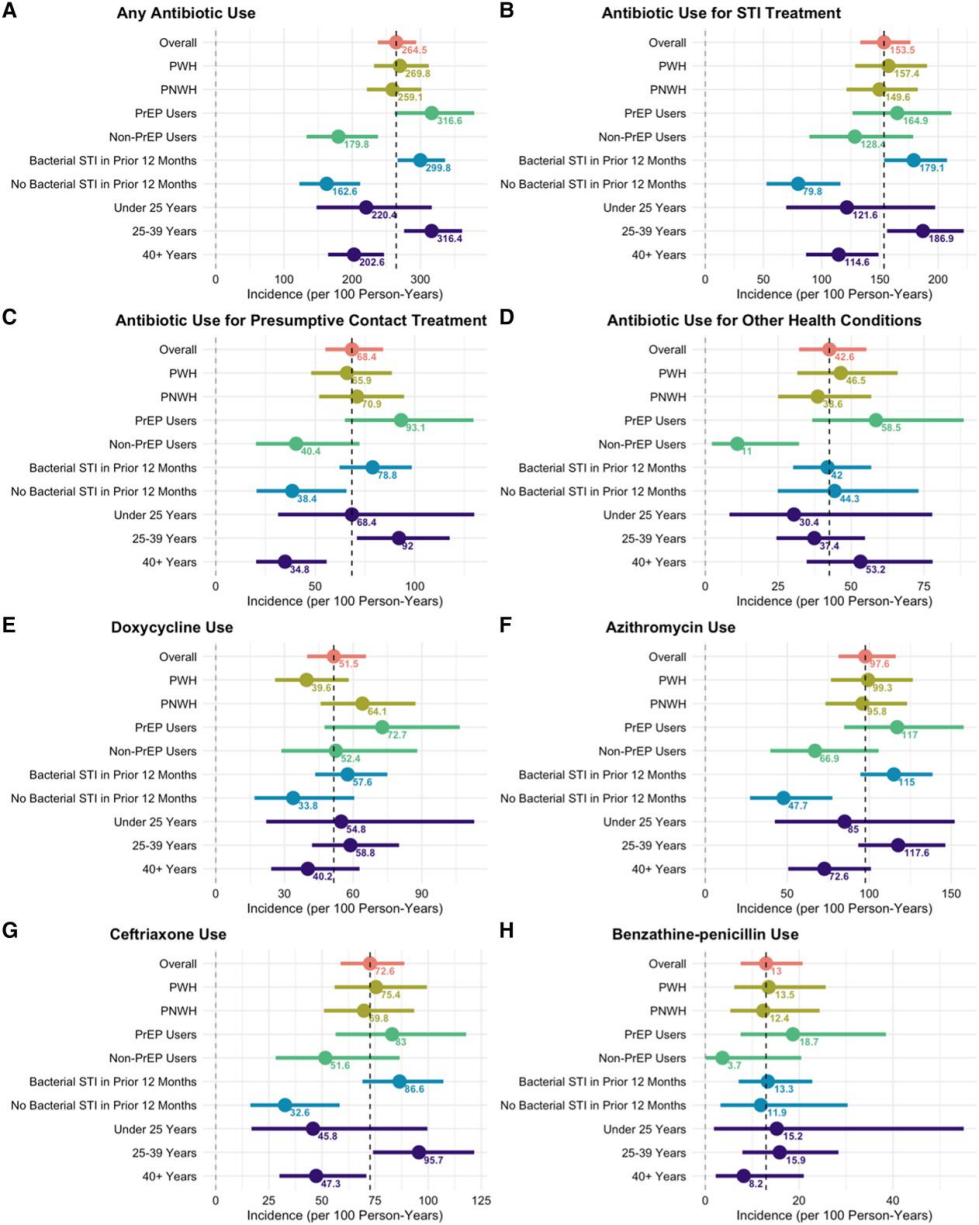
Incidence rates of antibiotic initiation overall, by treatment reason, and by antibiotic type in the ExGen Cohort, stratified by human immunodeficiency virus status and preexposure prophylaxis use at enrollment, reported sexually transmitted infection diagnosis within 12 months prior to enrollment, and age (<25, 25–39, ≥40 years). Abbreviations: PrEP, human immunodeficiency virus preexposure prophylaxis; PWH, people living with human immunodeficiency virus; PWOH, people living without human immunodeficiency virus; STI, sexually transmitted infection.

In primary analyses, there were 348 unique events of antibiotic initiation, resulting in an overall IR of 264.5 events per 100 PY (Table 2; Figure 2). STI treatment yielded the highest rate for antibiotic initiation (153.5 per 100 PY), followed by epidemiologic treatment for an STI contact (68.4 per 100 PY) and other health conditions (42.6 per 100 PY). Azithromycin (97.6 per 100 PY) and ceftriaxone (72.6 per 100 PY) were the most initiated antibiotics (Figure 2). In secondary analyses (Supplementary Tables 2*A–J*), unadjusted incidence of any antibiotic initiation did not differ in people living with versus without HIV (269.8 vs 259.1 per 100 PY; *P* = .71); however, initiation was significantly higher among PrEP versus non-PrEP users (316.6 vs 179.8 per 100 PY; *P* < .001).

**Table 2.** Incidence of Antibiotic Initiation and Days of Antibiotic Use Among Study Participants in the ExGen Study, 2016–2018 (N = 140)

Outcome	Cumulative Use^b^, No. (%)	Antibiotic Initiation		Days of Antibiotic Use^a^	
		No. of Events	Incidence Rate^c^ (95% CI)	No. of Days	Incidence Rate^c,d^ (95% CI)
Any antibiotic initiation	96 (69)	348	264.5 (237.5–293.8)	1696	1289.1 (1228.5–1352.0)
Reason for antibiotic initiation					
STI treatment	70 (50)	202	153.5 (133.1–176.2)	462	351.2 (319.9–384.7)
Epidemiologic treatment for STI contact	42 (30)	90	68.4 (55.0–84.1)	287	218.1 (193.6–244.9)
Other health condition treatment	26 (19)	56	42.6 (32.2–55.3)	947	719.8 (674.7–767.2)
Antibiotic-specific initiation					
Doxycycline	44 (31)	66	51.5 (39.9–65.6)	985	769.3 (722.0–818.9)
Azithromycin	71 (51)	126	97.6 (81.3–116.2)	146	113.1 (95.5–133.0)
Ceftriaxone	63 (45)	95	72.6 (58.8–88.8)	95	72.6 (58.8–88.8)
Benzathine-penicillin	14 (10)	17	13.0 (7.5–20.7)	17	13.0 (7.5–20.7)

Abbreviations: CI, confidence interval; STI, sexually transmitted infection.

^a^Reported days of antibiotic use for upper bound only; lower-bound estimates provided in the Supplementary Tables.

^b^Cumulative number of study participants reporting antibiotic use, among 140 total participants.

^c^Incidence per 100 person-years at risk.

^d^Two study participants contributed >500 days of antibiotic use (doxycycline and/or minocycline) for the treatment of acne and other chronic skin conditions. If these contributions were removed, the following “Days of antibiotic use” estimates would change: any antibiotic initiation, 1171 days, incidence rate (IR): 890.1 (95% CI, 839.8–942.6); other health condition treatment, 422 days, IR: 320.8 (95% CI, 290.9–352.9); doxycycline, 565 days, IR: 437.3 (95% CI, 402.0–474.9).

### Duration of Antibiotic Use

Study participants reported using antibiotics during 1696 total days of follow-up, resulting in an overall IR of 1289.1 days (3.53 years) of antibiotic use per 100 PY (Table 2). STI treatment was reported among 50% of all study participants, yet only accounted for 462 days of total use. Overall, doxycycline (985 days; IR, 769.3 days per 100 PY) and azithromycin (146 days; IR, 113.1 days per 100 PY) were the most frequently used antibiotics in the cohort (Table 2). Lower-bound estimates, including those for any antibiotic (1597 days; IR, 1213.9 days per 100 PY) or for other health conditions (848 days; IR, 644.6 days per 100 PY) were similar to upper-bound estimates. Despite only 19% of individuals reporting antibiotic treatment for other health conditions not specific to STIs, treatment for other conditions contributed most to overall duration of use, accounting for 56% of all days of antibiotic use (947 days; IR, 719.8 days per 100 PY). These conditions included acne and chronic skin infections (511 days), respiratory or ear/nose/throat infections (202 days), and acute skin and soft tissue infections (126 days), among others (Supplementary Table 3). Importantly, 2 individuals contributed >500 days of antibiotic use for the treatment of various skin conditions. If these contributions were removed, the IRs for overall use (890.1), treatment of other health conditions (320.8), and doxycycline (437.3) would decrease substantially (Table 2). Results of secondary analyses are presented in Supplementary Tables 2*A–J*.

### Factors Associated With Antibiotic Initiation

In univariate models, HIV status and PrEP use, age, race/ethnicity, and history of bacterial STI <12 months prior to enrollment were associated with antibiotic initiation and included in the multivariate model (Table 3). In the final multivariate model, age 25–39 years (IRR, 1.54 [95% confidence interval {CI}, 1.02–2.32]) and a history of bacterial STIs <12 months prior to enrollment (IRR, 1.81 [95% CI, 1.12–2.93]) were significantly associated with higher incidence of antibiotic initiation. HIV-positive status, PrEP use among people without HIV, and non-Hispanic Black/African American race were associated with antibiotic initiation; however, these associations were not significant at α ≤.05. In secondary analysis assessing antibiotic initiation for STI treatment only, age, race/ethnicity, and history of bacterial STI <12 months prior to enrollment were associated with antibiotic initiation and included in the multivariate model (Supplementary Table 4). In the final model, only a history of bacterial STIs was significantly associated with higher incidence of antibiotic initiation for STI treatment (IRR, 2.18 [95% CI, 1.16–4.11]).

**Table 3.** Factors Associated With Antibiotic Initiation in the ExGen Study, 2016–2018

Predictors	Univariate Analysis^a^		Multivariate Analysis^a^	
	IRR (95% CI)	*P* Value	IRR (95% CI)	*P* Value
HIV/PrEP status				
Living without HIV, not on PrEP	1.00		1.00	
Living without HIV, on PrEP	1.76 (1.00–3.09)	.05	1.65 (.95–2.87)	.08
Living with HIV	1.50 (.88–2.57)	.14	1.68 (.98–2.88)	.06
Age group				
≥40 y	1.00		1.00	
25–39 y	1.56 (1.04–2.35)	.03	1.54 (1.02–2.32)	.04
18–24 y	1.09 (.56–2.12)	.80	1.24 (.62–2.49)	.54
History of chronic medical conditions^b^	1.12 (.77–1.64)	.56	…	
Reported methamphetamine use^c^	0.88 (.58–1.33)	.53	…	
Race/ethnicity				
Non-Hispanic White	1.00		1.00	
Hispanic/Latinx	0.92 (.58–1.46)	.71	0.83 (.53–1.32)	.43
Non-Hispanic Black/African American	0.61 (.35–1.05)	.08	0.63 (.36–1.12)	.12
Another race/ethnicity	0.55 (.27–1.11)	.09	0.63 (.32–1.25)	.19
History of bacterial STI diagnosis <12 mo prior to enrollment^d^	1.84 (1.15–2.96)	.01	1.81 (1.12–2.93)	.02

Abbreviations: CI, confidence interval; HIV, human immunodeficiency virus; IRR, incidence rate ratio; PrEP, preexposure prophylaxis; STI, sexually transmitted infection.

^a^Implemented negative binomial regression with generalized estimating equations.

^b^Compared to no history of chronic medical conditions.

^c^Compared to no reported methamphetamine use.

^d^Compared to no history of bacterial STI diagnosis <12 months prior to enrollment.

## DISCUSSION

To our knowledge, this is the first study to characterize longitudinal antibiotic consumption among a cohort of MSM who would likely be eligible to receive doxy-PEP. We found that initiation of antibiotics during the 49 weeks of follow-up was very high, with 69% of 140 participants taking antibiotics at least once, resulting in 348 unique events of antibiotic initiation and 1696 days of use. One in 5 participants initiated 5 or more regimens for any reason during follow-up, and half of participants used antibiotics specifically for STI treatment.

The frequency of antibiotic use we observed was higher than results from other published studies. In a Vietnam-based study, 38% of 207 MSM reported using antibiotics in the previous 6 months, including 16% reporting use in the previous month [22]. In Peru, 41% of 386 MSM and transgender women presenting at sexual health clinics reported using any antibiotics in the last 3 months [23]. Even among MSM with diagnosed genital, anorectal, or pharyngeal GC in Belgium, only 43% of 42 participants reported any antibiotic use in the previous 12 months [24]. There are several potential reasons for higher levels of use observed in our cohort. First, study populations likely had differential access to antibiotics and other healthcare services, given substantial variability by geography. ExGen participants had access to frequent STI testing at urogenital and extragenital sites, which would drive increased diagnoses and subsequent treatments. Second, ExGen was designed to enroll participants who were likely to acquire bacterial STIs. Because many of the eligibility criteria are associated with antibiotic consumption, our cohort may have been more predisposed to antibiotic use than the general MSM population. However, this is also reflective of the population recommended to receive doxy-PEP by the CDC [7]. Third, there is lack of consistency in how antibiotic consumption is defined across studies. Most studies did not specify the reasons for use, which may have resulted in underreporting of antibiotics used to treat health conditions other than STIs.

An important finding in our study was the substantial contributions of other health conditions to the overall number of days of antibiotics used (947 of 1696 total days). Study participants cited treatment for various respiratory infections, including strep throat and pneumonia, and acute or chronic skin infections. Doxycycline use in our cohort was high (985 days), largely driven by the treatment of acne and other chronic skin conditions (406 days) among 2 individuals. Long-term systemic use of doxycycline for acne is not clinically recommended, suggesting that this pattern of use may have limited implications at the population level [25]. However, MSM, including people with HIV, have increased risk for several health conditions, including pneumococcal disease, *Staphylococcus aureus*, shigellosis, and giardiasis, and would be more likely to use antibiotics for the treatment of these conditions [26–29]. Therefore, a comprehensive approach to characterizing risk of antibiotic resistance among MSM should include considerations beyond treatment for common STIs.

Although determinants of antibiotic use have been well-described among the general population, little is known about the factors associated with increased antibiotic use among MSM [30]. In multivariate analyses, we found that MSM with a history of bacterial STIs within the previous 12 months and those aged 25–39 years had high rates of antibiotic initiation. Our findings provide important context for informing evidence-based conversations between clinicians and patients, given guidelines on doxy-PEP published by the CDC in June 2024 that recommend clinician-led counseling on the potential benefits, harms, and risk of antibiotic resistance [7].

An important consideration surrounding doxy-PEP implementation is the potential for subsequent increases in antibiotic consumption among MSM. The magnitude of this change is likely dependent on several factors, including underlying frequency and reasons for use. Although about 10% of the cohort initiated 5 or more regimens for STI treatment during follow-up, approximately 50% and approximately 10% of the study population initiated either 0 or 1 regimen for STI treatment, respectively. As many treatment regimens for STIs use single-dose approaches, doxy-PEP implementation in these individuals with low underlying antibiotic use could lead to increased antibiotic consumption among the broader MSM population. A recent modeling study found that, to balance doxy-PEP consumption with averted doxycycline doses for CT treatment in the United States, doxy-PEP would need to be restricted to individuals with an incidence of 7.8 infections per PY [31]. One individual in the ExGen cohort met this criterion. Future studies assessing doxy-PEP effectiveness and uptake among MSM should evaluate how doxy-PEP use increases or decreases overall antibiotic consumption and whether any potential increases in antibiotic resistance are primarily influenced by a larger population of individuals using antibiotics or by a smaller group of individuals who use antibiotics frequently.

There are notable strengths to our study. The ExGen Study was a prospective cohort study with 49 weeks of follow-up per participant, allowing us to observe longitudinal antibiotic use behaviors. Additionally, ExGen utilized medical record–based data to supplement and validate self-reported use, ensuring that nearly all antibiotic use among study participants was captured.

Our study also has limitations. First, we relied on self-reported antibiotic use, though patient medical records were used to validate and improve accuracy of self-reported measures, when possible. Second, several factors, including methamphetamine use and HIV status, are stigmatized and could be underreported. We attempted to minimize bias by using consistent, inclusive language in weekly surveys that reflected the identities and behaviors of our participants. Third, ExGen enrolled individuals who accessed sexual health services and were at increased risk for acquiring extragenital STIs. Findings from this analysis may not be generalizable to the broader MSM population in the United States or other settings. However, this population would be eligible for doxy-PEP and is therefore an important subgroup in which to assess antibiotic use.

In conclusion, most antibiotic regimens were initiated for STI-related conditions, whereas the largest proportion of days of antibiotic use were due to treatment of non-STI conditions. Our analysis provides a necessary foundation for assessing how doxy-PEP and changing standards of STI screening and treatment might affect antibiotic consumption among a target population for doxy-PEP. Future work is needed to understand antibiotic use among broader populations of MSM and surveillance of microorganisms potentially impacted by shifting patterns of STI-related antibiotic use, such as *Staphylococcus aureus*, should also be considered.

## Supplementary Material

ofaf051_Supplementary_Data

## Supplementary Data

Supplementary materials are available at *Open Forum Infectious Diseases* online. Consisting of data provided by the authors to benefit the reader, the posted materials are not copyedited and are the sole responsibility of the authors, so questions or comments should be addressed to the corresponding author.

## Notes

***Author contributions.*** All authors made substantial contributions to the conceptualization, analysis, synthesis, and/or write-up of this work.

***Acknowledgments.*** We thank our study participants and the Public Health–Seattle & King County Sexual Health Clinic for donating study space. We thank Micaela Haglund and Winnie Yeung for recruiting and coordinating with study participants, and Dr Julie Dombrowski for contributions to the conceptual development of this work.

***Patient consent.*** All enrolled study participants provided written informed consent for participation and medical record extraction. This study was approved by the University of Washington's Institutional Review Board (no. 50028).

***Financial support*.** The ExGen Study was funded by the National Institute of Allergy and Infectious Diseases (NIAID) of the National Institutes of Health (NIH) (award number K23 AI113185 to L. A. B.). This work was supported by the NIAID/NIH (grant number 1F31AI181431-01A1 to G. K. Z.). Specimen collection kits and test reagents were provided in-kind by Hologic Inc (San Diego, California). REDCap (data collection and management system) at the University of Washington's Institute for Translation Health Sciences is supported by the National Center for Advancing Translational Sciences of the NIH (award number UL1 TR002319). This work was additionally supported by the University of Washington/Fred Hutch Center for AIDS Research, an NIH-funded program under award AI027757, which is supported by the following institutes and centers: NIAID, National Cancer Institute, National Institute of Mental Health, National Institute on Drug Abuse, Eunice Kennedy Shriver National Institute of Child Health and Human Development, National Heart, Lung, and Blood Institute, National Institute on Aging, National Institute of General Medical Sciences, and National Institute of Diabetes and Digestive and Kidney Diseases.
