# Supplementary material for: High Incidence and Duration of Antibiotic Use Among a Cohort of Men Who Have Sex With Men in Seattle, Washington
Source: Open Forum Infect Dis. 2025 Jan 30;12(2):ofaf051. doi: 10.1093/ofid/ofaf051 (PMC11811903; doi:10.1093/ofid/ofaf051)
Supplement: ofaf051_Supplementary_Data [file ofaf051_supplementary_data.docx]

**SUPPLEMENTARY MATERIALS**

**TITLE:** High Incidence and Duration of Antibiotic Use Among a Cohort of Men Who Have Sex with Men in Seattle, WA

**TABLE OF CONTENTS:**

**Table S1:** *Reported Antibiotic Regimens: Durations & Clinically Relevant Buffer Periods Post-Regimen Completion* …………………………………...…..…………….…………………………**2**

**Table S2A:** *Secondary Analysis - Incidence of Antibiotic Initiation & Days of Antibiotic Use Among All Study Participants in the ExGen Study, 2016-2018 (N = 140) ...*………….…………**4**

**Table S2B:** *Secondary Analysis - Incidence of Antibiotic Initiation & Days of Antibiotic Use Among Study Participants Living with HIV in the ExGen Study, 2016-2018 (N = 71)* ………….**5**

**Table S2C:** *Secondary Analysis - Incidence of Antibiotic Initiation & Days of Antibiotic Use Among Study Participants Not Living with HIV in the ExGen Study, 2016-2018 (N = 69)*..........**6**

**Table S2D:** *Secondary Analysis - Incidence of Antibiotic Initiation & Days of Antibiotic Use Among Study Participants on PrEP in the ExGen Study, 2016-2018 (N = 40)* …………….……**7**

**Table S2E:** *Secondary Analysis - Incidence of Antibiotic Initiation & Days of Antibiotic Use Among Study Participants Not Living with HIV, Not on PrEP in the ExGen Study, 2016-2018 (N = 29)*………………………………………………………………………………………………...**8**

**Table S2F:** *Secondary Analysis - Incidence of Antibiotic Initiation & Days of Antibiotic Use Among Study Participants with a Bacterial STI Diagnosis 12 Months Prior to Enrollment in the ExGen Study, 2016-2018 (N = 104)* ………………...…………………………………………...…**9**

**Table S2G:** *Secondary Analysis - Incidence of Antibiotic Initiation & Days of Antibiotic Use Among Study Participants without a Bacterial STI Diagnosis 12 Months Prior to Enrollment in the ExGen Study, 2016-2018 (N = 36)* ……………………..……………..………………………**10**

**Table S2H:** *Secondary Analysis - Incidence of Antibiotic Initiation & Days of Antibiotic Use Among Study Participants Under 25 Years of Age in the ExGen Study, 2016-2018 (N = 14)* …………………………………………………………………………………………………..……**11**

**Table S2I:** *Secondary Analysis - Incidence of Antibiotic Initiation & Days of Antibiotic Use Among Study Participants 25 – 39 Years of Age in the ExGen Study, 2016-2018 (N = 74)* …**12**

**Table S2J:** *Secondary Analysis - Incidence of Antibiotic Initiation & Days of Antibiotic Use Among Study Participants 40+ Years of Age in the ExGen Study, 2016-2018 (N = 52)* ……...**13**

**Table S3:** *Days of Antibiotic Use for the Treatment of Other Health Conditions Among Study Participants in the ExGen Study, 2016-2018……………………………………………...*……...**14**

**Table S4:** *Factors Associated with Antibiotic Initiation for STI Treatment in the ExGen Study, 2016-2018* ……………………………………..…………………………………………………… **15**

| **Table S1. Reported Antibiotic Regimens: Durations & Clinically Relevant Buffer Periods Post-Regimen Completion^a^** | | | | |
| --- | --- | --- | --- | --- |
| Reported Clinical Diagnosis (Antibiotic) |  | Regimen Duration^b^ |  | Buffer Period^c^ |
| *Chlamydia trachomatis* |  |  |  |  |
| Azithromycin |  | 1 Day |  | + 6 Days |
| Doxycycline |  | 7 Days |  | + 5 Days |
| *Neisseria gonorrhoeae* |  |  |  |  |
| Cefixime, Ceftriaxone, Gentamicin |  | 1 Day |  | + 2 Days |
| Azithromycin |  | 1 Day |  | + 6 Days |
| Syphilis |  |  |  |  |
| Benzathine-penicillin^d^, Penicillin |  | 1 Day (per shot) |  | + 6 Days |
| Doxycycline |  | 14 Days |  | + 5 Days |
| Non-Gonococcal Urethritis |  |  |  |  |
| Doxycycline |  | 7 Days |  | + 5 Days |
| Urinary Tract Infection |  |  |  |  |
| Ciprofloxacin |  | 3 – 7 Days |  | + 2 Days |
| Moxifloxacin |  | 5 – 7 Days |  | + 2 Days |
| Epididymitis |  |  |  |  |
| Doxycycline, Ofloxacin |  | 10 Days |  | + 2 Days |
| Proctitis |  |  |  |  |
| Doxycycline |  | 21 Days |  | + 5 Days |
| Anal Fissure |  |  |  |  |
| Doxycycline |  | 7 Days |  | + 2 Days |
| Respiratory Infection |  |  |  |  |
| Azithromycin |  | 3 – 5 Days |  | + 2 Days |
| Doxycycline, Levofloxacin |  | 5 Days |  | + 2 Days |
| Bactrim |  | 21 Days |  | + 0 Days |
| Strep Throat |  |  |  |  |
| Amoxicillin, Cephalexin |  | 10 Days |  | + 2 Days |
| Pneumonia |  |  |  |  |
| Cephalexin |  | 5 – 7 Days |  | + 2 Days |
| Bronchitis |  |  |  |  |
| Ciprofloxacin |  | 5 – 7 Days |  | + 2 Days |
| Skin Infection or Abscess (Acute) |  |  |  |  |
| Vancomycin |  | 3 – 7 Days |  | + 0 Days |
| Bactrim, Cephalexin, Mupirocin |  | 5 – 7 Days |  | + 2 Days |
| Doxycycline |  | 5 – 14 Days |  | + 2 Days |
| Skin Infection or Acne (Chronic) |  |  |  |  |
| Doxycycline, Minocycline Hydrochloride |  | Duration of Reporting |  | NA |
| Shigella |  |  |  |  |
| Ciprofloxacin |  | 3 – 7 Days |  | + 2 Days |
| Giardia |  |  |  |  |
| Tinidazole |  | 1 Day |  | + 7 Days |
| Tooth Abscess |  |  |  |  |
| Penicillin |  | 7 – 14 Days |  | + 0 Days |
| ^a^ Antibiotic regimens for non-STI specific conditions were most often patient reported and may not align with standard treatment practices. In instances where non-standard use was reported, we estimated potential duration and buffer periods informed by clinical practice and patient reported information.  ^b^ Duration of antibiotic regimen, dependent on clinical diagnosis. Upper and lower bounds provided for antibiotics regimens with potential ranges in duration.  ^c^ Clinically relevant time period post-regimen where study participants would not be prescribed the antibiotic again. Time excluded from antibiotic-specific person-time at risk.  ^d^ Also referred to as Bicillin L-A. | | | | |

| **Table S2A. Secondary Analysis - Incidence of Antibiotic Initiation & Days of Antibiotic Use Among All Study Participants in the ExGen Study, 2016-2018 (N = 140)** | | | | | | | |
| --- | --- | --- | --- | --- | --- | --- | --- |
|  |  | Antibiotic Initiation | |  | Days of Antibiotic Use | |  |
| Outcome | Cumulative Use^a^  N (%) | # of Events | Incidence Rate^b,c^  (95% CI) |  | # of Days | Incidence Rate^b,d,e^  (95% CI) |  |
| Any Antibiotic Initiation | 96 (69) | 348 | UB: 264.5 (237.5, 293.8)  LB: NA |  | UB: 1696  LB: 1597 | UB: 1289.1 (1228.5, 1352.0)  LB: 1213.9 (1155.1, 1274.9) |  |
| Reason for Antibiotic Initiation |  |  |  |  |  |  |  |
| STI Treatment | 70 (50) | 202 | UB: 153.5 (133.1, 176.2)  LB: NA |  | UB: 462  LB: NA | UB: 351.2 (319.9, 384.7)  LB: NA |  |
| Epidemiologic Treatment for STI Contact | 42 (30) | 90 | UB: 68.4 (55.0, 84.1)  LB: NA |  | UB: 287  LB: NA | UB: 218.1 (193.6, 244.9)  LB: NA |  |
| Other Health Condition Treatment | 26 (19) | 56 | UB: 42.6 (32.2, 55.3)  LB: NA |  | UB: 947  LB: 848 | UB: 719.8 (674.7, 767.2)  LB: 644.6 (601.9, 689.4) |  |
| Antibiotic-specific Initiation |  |  |  |  |  |  |  |
| Doxycycline | 44 (31) | 66 | UB: 51.5 (39.9, 65.6)  LB: 51.5 (39.8, 65.5) |  | UB: 985  LB: 956 | UB: 769.3 (722.0, 818.9)  LB: 746.2 (699.6, 795.0) |  |
| Azithromycin | 71 (51) | 126 | UB: 97.6 (81.3, 116.2)  LB: 97.6 (81.3, 116.2) |  | UB: 146  LB: 140 | UB: 113.1 (95.5, 133.0  LB: 108.4 (91.2, 127.9) |  |
| Ceftriaxone | 63 (45) | 95 | UB: 72.6 (58.8, 88.8)  LB: NA |  | UB: 95  LB: NA | UB: 72.6 (58.8, 88.8)  LB: NA |  |
| Benzathine-penicillin | 14 (10) | 17 | UB: 13.0 (7.5, 20.7)  LB: NA |  | UB: 17  LB: NA | UB: 13.0 (7.5, 20.7)  LB: NA |  |
| ^a^ Cumulative number of study participants reporting antibiotic use during follow-up, among 140 total participants  ^b^ Incidence per 100 person-years at risk  ^c^ Upper- and lower-bound estimates for incidence of antibiotic initiation incorporate removal of person-time at risk based on duration ranges for antibiotic regimens outlined in Supplemental Table 1.  ^d^ Upper- and lower-bound estimates for days of antibiotic use are based on ranges for antibiotic regimens outlined in Supplemental Table 1. For estimates without a range of possible regimen durations, the lower bound estimate is listed as NA.  ^e^ Two study participants contributed over 500 days of antibiotic use (doxycycline and/or minocycline) for the treatment of acne and other chronic skin conditions. If these contributions were removed, the following “Days of Antibiotic Use” estimates would change: Upper Bound - *Any Antibiotic Initiation*, 1171 days, IR: 890.1 (839.8, 942.6); Other Health Condition Treatment, 422 days, IR: 320.8 (290.9, 352.9); Doxycycline, 565 days, IR: 437.3 (402.0, 474.9). Lower Bound - *Any Antibiotic Initiation*, 1081 days, IR: 821.7 (773.4, 872.1); Other Health Condition Treatment, 332 days, IR: 252.4 (225.9, 281.0); Doxycycline, 545 days, IR: 421.7 (387.0, 458.6)  Abbreviations: CI, confidence interval; STI, sexually transmitted infection; UB, upper bound; LB, lower bound; NA, not applicable | | | | | | | |

| **Table S2B. Secondary Analysis - Incidence of Antibiotic Initiation & Days of Antibiotic Use Among Study Participants Living with HIV in the ExGen Study, 2016-2018 (N = 71)** | | | | | | |
| --- | --- | --- | --- | --- | --- | --- |
|  |  | Antibiotic Initiation | |  | Days of Antibiotic Use | |
| Outcome | Cumulative Use^a^  N (%) | # of Events | Incidence Rate^b,c^  (95% CI) |  | # of Days | Incidence Rate^b,d^  (95% CI) |
| Any Antibiotic Initiation | 49 (69) | 180 | UB: 269.8 (231.8, 312.2)  LB: NA |  | UB: 747  LB: 677 | UB: 1119.6 (1040.7, 1202.8)  LB: 1014.7 (939.7, 1094.1) |
| Reason for Antibiotic Initiation |  |  |  |  |  |  |
| STI Treatment | 37 (52) | 105 | UB: 157.4 (128.7, 190.5)  LB: NA |  | UB: 216  LB: NA | UB: 323.7 (282.0, 369.9)  LB: NA |
| Epidemiologic Treatment for STI Contact | 19 (27) | 44 | UB: 65.9 (47.9, 88.5)  LB: NA |  | UB: 113  LB: NA | UB: 169.4 (139.6, 203.6)  LB: NA |
| Other Health Condition Treatment | 12 (17) | 31 | UB: 46.5 (31.6, 65.9)  LB: NA |  | UB: 418  LB: 348 | UB: 626.5 (567.9, 689.5)  LB: 521.6 (468.2, 579.4) |
| Antibiotic-specific Initiation |  |  |  |  |  |  |
| Doxycycline | 20 (28) | 26 | UB: 39.6 (25.9, 58.1)  LB: 39.6 (25.9, 58.0) |  | UB: 294  LB: 276 | UB: = 448.2 (398.5, 502.5)  LB: = 420.5 (372.3, 473.1) |
| Azithromycin | 38 (54) | 65 | UB: 99.3 (76.6, 126.5)  LB: 99.3 (76.6, 126.5) |  | UB: 73  LB: 69 | UB: 111.5 (87.4, 140.2)  LB: 105.4 (82.0, 133.3) |
| Ceftriaxone | 32 (45) | 50 | UB: 75.4 (56.0, 99.4)  LB: NA |  | UB: 50  LB: NA | UB: 75.4 (56.0, 99.4)  LB: NA |
| Benzathine-penicillin | 8 (11) | 9 | UB: 13.5 (6.2, 25.7)  LB: NA |  | UB: 9  LB: NA | UB: 13.5 (6.2, 25.7)  LB: NA |
| ^a^ Cumulative number of study participants reporting antibiotic use during follow-up, among 71 participants  ^b^ Incidence per 100 person-years at risk  ^c^ Upper- and lower-bound estimates for incidence of antibiotic initiation incorporate removal of person-time at risk based on duration ranges for antibiotic regimens outlined in Supplemental Table 1.  ^d^ Upper- and lower-bound estimates for days of antibiotic use are based on ranges for antibiotic regimens outlined in Supplemental Table 1. For estimates without a range of possible regimen durations, the lower bound estimate is listed as NA.  Abbreviations: CI, confidence interval; STI, sexually transmitted infection; UB, upper bound; LB, lower bound; NA, not applicable | | | | | | |

| **Table S2C. Secondary Analysis - Incidence of Antibiotic Initiation & Days of Antibiotic Use Among Study Participants Not Living with HIV in the ExGen Study, 2016-2018 (N = 69)** | | | | | | |
| --- | --- | --- | --- | --- | --- | --- |
|  |  | Antibiotic Initiation | |  | Days of Antibiotic Use | |
| Outcome | Cumulative Use^a^  N (%) | # of Events | Incidence Rate^b,c^  (95% CI) |  | # of Days | Incidence Rate^b,d^  (95% CI) |
| Any Antibiotic Initiation | 47 (68) | 168 | UB: 259.1 (221.4, 301.4)  LB: NA |  | UB: 949  LB: 920 | UB: 1463.6 (1371.9, 1559.7)  LB: 1418.9 (1328.6, 1513.6) |
| Reason for Antibiotic Initiation |  |  |  |  |  |  |
| STI Treatment | 33 (48) | 97 | UB: 149.6 (121.3, 182.5)  LB: NA |  | UB: 246  LB: NA | UB: 379.4 (333.5, 429.9)  LB: NA |
| Epidemiologic Treatment for STI Contact | 23 (33) | 46 | UB: 70.9 (51.9, 94.6)  LB: NA |  | UB: 174  LB: NA | UB: 268.3 (230.0, 311.3)  LB: NA |
| Other Health Condition Treatment | 14 (20) | 25 | UB: 38.6 (25.0, 56.9)  LB: NA |  | UB: 529  LB: 500 | UB: 815.8 (747.8, 888.4)  LB: 771.1 (705.0, 841.8) |
| Antibiotic-specific Initiation |  |  |  |  |  |  |
| Doxycycline | 24 (35) | 40 | UB: 64.1 (45.8, 87.2)  LB: 64.0 (45.7, 87.2) |  | UB: 691  LB: 680 | UB: 1106.5 (1025.6, 1192.2)  LB: 1088.4 (1008.1, 1173.4) |
| Azithromycin | 33 (48) | 61 | UB: 95.8 (73.3, 123.1)  LB: 95.8 (73.3, 123.1) |  | UB: 73  LB: 71 | UB: 114.7 (89.9, 144.2)  LB: 111.5 (87.1, 140.7) |
| Ceftriaxone | 31 (45) | 45 | UB: 69.8 (50.9, 93.4)  LB: NA |  | UB: 45  LB: NA | UB: 69.8 (50.9, 93.4)  LB: NA |
| Benzathine-penicillin | 6 (9) | 8 | UB: 12.4 (5.3, 24.4)  LB: NA |  | UB: 8  LB: NA | UB: 12.4 (5.3, 24.4)  LB: NA |
| ^a^ Cumulative number of study participants reporting antibiotic use during follow-up, among 69 total participants  ^b^ Incidence per 100 person-years at risk  ^c^ Upper- and lower-bound estimates for incidence of antibiotic initiation incorporate removal of person-time at risk based on duration ranges for antibiotic regimens outlined in Supplemental Table 1.  ^d^ Upper- and lower-bound estimates for days of antibiotic use are based on ranges for antibiotic regimens outlined in Supplemental Table 1. For estimates without a range of possible regimen durations, the lower bound estimate is listed as NA.  Abbreviations: CI, confidence interval; STI, sexually transmitted infection; UB, upper bound; LB, lower bound; NA, not applicable | | | | | | |

| **Table S2D. Secondary Analysis - Incidence of Antibiotic Initiation & Days of Antibiotic Use Among Study Participants on PrEP in the ExGen Study, 2016-2018 (N = 40)** | | | | | | |
| --- | --- | --- | --- | --- | --- | --- |
|  |  | Antibiotic Initiation | |  | Days of Antibiotic Use | |
| Outcome | Cumulative Use^a^  N (%) | # of Events | Incidence Rate^b,c^  (95% CI) |  | # of Days | Incidence Rate^b,d^  (95% CI) |
| Any Antibiotic Initiation | 30 (75) | 119 | UB: 316.6 (262.3, 378.8)  LB: NA |  | UB: 769  LB: 746 | UB: 2045.8 (1903.8, 2195.7)  LB: 1984.6 (1844.7, 2132.3) |
| Reason for Antibiotic Initiation |  |  |  |  |  |  |
| STI Treatment | 20 (50) | 62 | UB: 164.9 (126.5, 211.4)  LB: NA |  | UB: 143  LB: NA | UB: 380.4 (320.6, 448.1)  LB: NA |
| Epidemiologic Treatment for STI Contact | 17 (43) | 35 | UB: 93.1 (64.9, 129.5)  LB: NA |  | UB: 118  LB: NA | UB: 313.9 (259.8, 375.9)  LB: NA |
| Other Health Condition Treatment | 11 (28) | 22 | UB: 58.5 (36.7, 88.6)  LB: NA |  | UB: 508  LB: 485 | UB: 1351.5 (1236.5, 1474.3)  LB: 1290.3 (1178.0, 1410.4) |
| Antibiotic-specific Initiation |  |  |  |  |  |  |
| Doxycycline | 15 (38) | 26 | UB: 72.7 (47.5, 106.6)  LB: 72.7 (47.5, 106.5) |  | UB: 558  LB: 547 | UB: 1560.8 (1434.0, 1695.9)  LB: 1528.8 (1403.3, 1662.4) |
| Azithromycin | 23 (58) | 43 | UB: 117.0 (84.7, 157.6)  LB: 117.0 (84.7, 157.6) |  | UB: 55  LB: 53 | UB: 149.6 (112.7, 194.8)  LB: 144.2 (108.0, 188.6) |
| Ceftriaxone | 22 (55) | 31 | UB: 83.0 (56.4, 117.9)  LB: NA |  | UB: 31  LB: NA | UB: 83.0 (56.4, 117.9)  LB: NA |
| Benzathine-penicillin | 5 (13) | 7 | UB: 18.7 (7.5, 38.5)  LB: NA |  | UB: 7  LB: NA | UB: 18.7 (7.5, 38.5)  LB: NA |
| ^a^ Cumulative number of study participants reporting antibiotic use during follow-up, among 40 total participants  ^b^ Incidence per 100 person-years at risk  ^c^ Upper- and lower-bound estimates for incidence of antibiotic initiation incorporate removal of person-time at risk based on duration ranges for antibiotic regimens outlined in Supplemental Table 1.  ^d^ Upper- and lower-bound estimates for days of antibiotic use are based on ranges for antibiotic regimens outlined in Supplemental Table 1. For estimates without a range of possible regimen durations, the lower bound estimate is listed as NA.  Abbreviations: CI, confidence interval; STI, sexually transmitted infection; UB, upper bound; LB, lower bound; NA, not applicable | | | | | | |

| **Table S2E. Secondary Analysis - Incidence of Antibiotic Initiation & Days of Antibiotic Use Among Study Participants Not Living with HIV, Not on PrEP in the ExGen Study, 2016-2018 (N = 29)** | | | | | | |
| --- | --- | --- | --- | --- | --- | --- |
|  |  | Antibiotic Initiation | |  | Days of Antibiotic Use | |
| Outcome | Cumulative Use^a^  N (%) | # of Events | Incidence Rate^b,c^  (95% CI) |  | # of Days | Incidence Rate^b,d^  (95% CI) |
| Any Antibiotic Initiation | 17 (59) | 49 | UB: 179.8 (133.0, 237.7)  LB: NA |  | UB: 180  LB: 174 | UB: 660.5 (567.5, 764.4)  LB: 638.5 (547.1, 740.7) |
| Reason for Antibiotic Initiation |  |  |  |  |  |  |
| STI Treatment | 13 (45) | 35 | UB: 128.4 (89.5, 178.6)  LB: NA |  | UB: 103  LB: NA | UB: 378.0 (308.5, 458.4)  LB: NA |
| Epidemiologic Treatment for STI Contact | 6 (21) | 11 | UB: 40.4 (20.1, 72.2)  LB: NA |  | UB: 56  LB: NA | UB: 205.5 (155.2, 266.8)  LB: NA |
| Other Health Condition Treatment | 3 (10) | 3 | UB: 11.0 (2.3, 32.2)  LB: NA |  | UB: 21  LB: 15 | UB: 77.1 (47.7, 117.8)  LB: 55.0 (30.8, 90.8) |
| Antibiotic-specific Initiation |  |  |  |  |  |  |
| Doxycycline | 9 (31) | 14 | UB: 52.4 (28.7, 88.0)  LB: 52.4 (28.7, 88.0) |  | UB: 133  LB: 133 | UB: 498.2 (417.1, 590.4)  LB: 498.2 (417.1, 590.4) |
| Azithromycin | 10 (34) | 18 | UB: 66.9 (39.6, 105.7)  LB: 66.9 (39.6, 105.7) |  | UB: 18  LB: 18 | UB: 66.9 (39.6, 105.7)  LB: 66.9 (39.6, 105.7) |
| Ceftriaxone | 9 (31) | 14 | UB: 51.6 (28.2, 86.6)  LB: NA |  | UB: 14  LB: NA | UB: 51.6 (28.2, 86.6)  LB: NA |
| Benzathine-penicillin | 1 (3) | 1 | UB: 3.7 (0.1, 20.5)  LB: NA |  | UB: 1  LB: NA | UB: 3.7 (0.1, 20.5)  LB: NA |
| ^a^ Cumulative number of study participants reporting antibiotic use during follow-up, among 29 total participants  ^b^ Incidence per 100 person-years at risk  ^c^ Upper- and lower-bound estimates for incidence of antibiotic initiation incorporate removal of person-time at risk based on duration ranges for antibiotic regimens outlined in Supplemental Table 1.  ^d^ Upper- and lower-bound estimates for days of antibiotic use are based on ranges for antibiotic regimens outlined in Supplemental Table 1. For estimates without a range of possible regimen durations, the lower bound estimate is listed as NA.  Abbreviations: CI, confidence interval; STI, sexually transmitted infection; UB, upper bound; LB, lower bound; NA, not applicable | | | | | | |

| **Table S2F. Secondary Analysis - Incidence of Antibiotic Initiation & Days of Antibiotic Use Among Study Participants with a Bacterial STI Diagnosis 12 Months Prior to Enrollment in the ExGen Study, 2016-2018 (N = 104)** | | | | | | |
| --- | --- | --- | --- | --- | --- | --- |
|  |  | Antibiotic Initiation | |  | Days of Antibiotic Use | |
| Outcome | Cumulative Use^a^  N (%) | # of Events | Incidence Rate^b,c^  (95% CI) |  | # of Days | Incidence Rate^b,d^  (95% CI) |
| Any Antibiotic Initiation | 77 (74) | 293 | UB: 299.8 (266.5, 336.2)  LB: NA |  | UB: 1111  LB: 1050 | UB: 1136.8 (1070.9, 1205.7)  LB: 1074.4 (1010.4, 1141.4) |
| Reason for Antibiotic Initiation |  |  |  |  |  |  |
| STI Treatment | 58 (56) | 175 | UB: 179.1 (153.5, 207.6)  LB: NA |  | UB: 389  LB: NA | UB: 398.0 (359.5, 439.6)  LB: NA |
| Epidemiologic Treatment for STI Contact | 36 (35) | 77 | UB: 78.8 (62.2, 98.5)  LB: NA |  | UB: 255  LB: NA | UB: 260.9 (229.9, 295.0)  LB: NA |
| Other Health Condition Treatment | 20 (19) | 41 | UB: 42.0 (30.1, 56.9)  LB: NA |  | UB: 467  LB: 406 | UB: 477.8 (435.5, 523.2)  LB: 415.4 (376.0, 457.9) |
| Antibiotic-specific Initiation |  |  |  |  |  |  |
| Doxycycline | 36 (35) | 55 | UB: 57.6 (43.4, 75.0)  LB: 57.6 (43.4, 74.9) |  | UB: 558  LB: 538 | UB: 584.3 (536.8, 634.8)  LB: 563.0 (516.4, 612.7) |
| Azithromycin | 60 (58) | 110 | UB: 115.0 (94.5, 138.7)  LB: 115.0 (94.5, 138.6) |  | UB: 126  LB: 120 | UB: 131.8 (109.8, 156.9)  LB: 125.5 (104.0, 150.0) |
| Ceftriaxone | 55 (53) | 84 | UB: 86.6 (69.0, 107.2)  LB: NA |  | UB: 84  LB: NA | UB: 86.6 (69.0, 107.2)  LB: NA |
| Benzathine-penicillin | 11 (11) | 13 | UB: 13.3 (7.1, 22.8)  LB: NA |  | UB: 13  LB: NA | UB: 13.3 (7.1, 22.8)  LB: NA |
| ^a^ Cumulative number of study participants reporting antibiotic use during follow-up, among 104 total participants  ^b^ Incidence per 100 person-years at risk  ^c^ Upper- and lower-bound estimates for incidence of antibiotic initiation incorporate removal of person-time at risk based on duration ranges for antibiotic regimens outlined in Supplemental Table 1.  ^d^ Upper- and lower-bound estimates for days of antibiotic use are based on ranges for antibiotic regimens outlined in Supplemental Table 1. For estimates without a range of possible regimen durations, the lower bound estimate is listed as NA.  Abbreviations: CI, confidence interval; STI, sexually transmitted infection; UB, upper bound; LB, lower bound; NA, not applicable | | | | | | |

| **Table S2G. Secondary Analysis - Incidence of Antibiotic Initiation & Days of Antibiotic Use Among Study Participants without a Bacterial STI Diagnosis 12 Months Prior to Enrollment in the ExGen Study, 2016-2018 (N = 36)** | | | | | | |
| --- | --- | --- | --- | --- | --- | --- |
|  |  | Antibiotic Initiation | |  | Days of Antibiotic Use | |
| Outcome | Cumulative Use^a^  N (%) | # of Events | Incidence Rate^b,c^  (95% CI) |  | # of Days | Incidence Rate^b,d^  (95% CI) |
| Any Antibiotic Initiation | 19 (53) | 55 | UB: 162.6 (122.5, 211.6)  LB: NA |  | UB: 585  LB: 547 | UB: 1729.2 (1591.9, 1875.2)  LB: 1616.9 (1484.2, 1758.3) |
| Reason for Antibiotic Initiation |  |  |  |  |  |  |
| STI Treatment | 12 (33) | 27 | UB: 79.8 (52.6, 116.1)  LB: NA |  | UB: 73  LB: NA | UB: 215.8 (169.1, 271.3)  LB: NA |
| Epidemiologic Treatment for STI Contact | 6 (17) | 13 | UB: 38.4 (20.5, 65.7)  LB: NA |  | UB: 32  LB: NA | UB: 94.6 (64.7, 133.5)  LB: NA |
| Other Health Condition Treatment | 6 (17) | 15 | UB: 44.3 (24.8, 73.1)  LB: NA |  | UB: 480  LB: 442 | UB: 1418.9 (1294.7, 1551.6)  LB: 1306.5 (1187.5, 1434.2) |
| Antibiotic-specific Initiation |  |  |  |  |  |  |
| Doxycycline | 8 (22) | 11 | UB: 33.8 (16.9, 60.5)  LB: 33.8 (16.9, 60.5) |  | UB: 427  LB: 418 | UB: 1312.6 (1191.0, 1443.2)  LB: 1283.9 (1163.8, 1413.1) |
| Azithromycin | 11 (31) | 16 | UB: 47.7 (27.3, 77.5)  LB: 47.7 (27.3, 77.5) |  | UB: 20  LB: 20 | UB: 59.7 (36.5, 92.2)  LB: 59.7 (36.5, 92.2) |
| Ceftriaxone | 8 (22) | 11 | UB: 32.6 (16.3, 58.3)  LB: NA |  | UB: 11  LB: NA | UB: 32.6 (16.3, 58.3)  LB: NA |
| Benzathine-penicillin | 3 (8) | 4 | UB: 11.9 (3.2, 30.3)  LB: NA |  | UB: 4  LB: NA | UB: 11.9 (3.2, 30.3)  LB: NA |
| ^a^ Cumulative number of study participants reporting antibiotic use during follow-up, among 36 total participants  ^b^ Incidence per 100 person-years at risk  ^c^ Upper- and lower-bound estimates for incidence of antibiotic initiation incorporate removal of person-time at risk based on duration ranges for antibiotic regimens outlined in Supplemental Table 1.  ^d^ Upper- and lower-bound estimates for days of antibiotic use are based on ranges for antibiotic regimens outlined in Supplemental Table 1. For estimates without a range of possible regimen durations, the lower bound estimate is listed as NA.  Abbreviations: CI, confidence interval; STI, sexually transmitted infection; UB, upper bound; LB, lower bound; NA, not applicable | | | | | | |

| **Table S2H. Secondary Analysis - Incidence of Antibiotic Initiation & Days of Antibiotic Use Among Study Participants Under 25 Years of Age in the ExGen Study, 2016-2018 (N = 14)** | | | | | | |
| --- | --- | --- | --- | --- | --- | --- |
|  |  | Antibiotic Initiation | |  | Days of Antibiotic Use | |
| Outcome | Cumulative Use^a^  N (%) | # of Events | Incidence Rate^b,c^  (95% CI) |  | # of Days | Incidence Rate^b,d^  (95% CI) |
| Any Antibiotic Initiation | 10 (71) | 29 | UB: 220.4 (147.6, 316.6)  LB: NA |  | UB: 238  LB: 229 | UB: 1809.0 (1586.5, 2054.1)  LB: 1740.6 (1522.5, 1981.3) |
| Reason for Antibiotic Initiation |  |  |  |  |  |  |
| STI Treatment | 7 (50) | 16 | UB: 121.6 (69.5, 197.5)  LB: NA |  | UB: 34  LB: NA | UB: 258.4 (179.0, 361.1)  LB: NA |
| Epidemiologic Treatment for STI Contact | 4 (29) | 9 | UB: 68.4 (31.3, 129.9)  LB: NA |  | UB: 21  LB: NA | UB: 159.6 (98.8, 244.0)  LB: NA |
| Other Health Condition Treatment | 2 (14) | 4 | UB: 30.4 (8.3, 77.8)  LB: NA |  | UB: 183  LB: 174 | UB: 1391.0 (1196.7, 1607.8)  LB: 1322.6 (1133.4, 1534.4) |
| Antibiotic-specific Initiation |  |  |  |  |  |  |
| Doxycycline | 4 (29) | 7 | UB: 54.8 (22.0, 112.9)  LB: 54.7 (22.0, 112.7) |  | UB: 112  LB: 103 | UB: 876.7 (721.9, 1054.9)  LB: 804.7 (656.8, 975.9) |
| Azithromycin | 7 (50) | 11 | UB: 85.0 (42.4, 152.0)  LB: 85.0 (42.4, 152.0) |  | UB: 11  LB: 11 | UB: 85.0 (42.4, 152.0)  LB: 85.0 (42.4, 152.0) |
| Ceftriaxone | 4 (29) | 6 | UB: 45.8 (16.8, 99.6)  LB: NA |  | UB: 6  LB: NA | UB: 45.8 (16.8, 99.6)  LB: NA |
| Benzathine-penicillin | 2 (14) | 2 | UB: 15.2 (1.8, 55.1)  LB: NA |  | UB: 2  LB: NA | UB: 15.2 (1.8, 55.1)  LB: NA |
| ^a^ Cumulative number of study participants reporting antibiotic use during follow-up, among 14 total participants  ^b^ Incidence per 100 person-years at risk  ^c^ Upper- and lower-bound estimates for incidence of antibiotic initiation incorporate removal of person-time at risk based on duration ranges for antibiotic regimens outlined in Supplemental Table 1.  ^d^ Upper- and lower-bound estimates for days of antibiotic use are based on ranges for antibiotic regimens outlined in Supplemental Table 1. For estimates without a range of possible regimen durations, the lower bound estimate is listed as NA.  Abbreviations: CI, confidence interval; STI, sexually transmitted infection; UB, upper bound; LB, lower bound; NA, not applicable | | | | | | |

| **Table S2I. Secondary Analysis - Incidence of Antibiotic Initiation & Days of Antibiotic Use Among Study Participants 25 – 39 Years of Age in the ExGen Study, 2016-2018 (N = 74)** | | | | | | |
| --- | --- | --- | --- | --- | --- | --- |
|  |  | Antibiotic Initiation | |  | Days of Antibiotic Use | |
| Outcome | Cumulative Use^a^  N (%) | # of Events | Incidence Rate^b,c^  (95% CI) |  | # of Days | Incidence Rate^b,d^  (95% CI) |
| Any Antibiotic Initiation | 55 (74) | 220 | UB: 316.4 (275.9, 361.0)  LB: NA |  | UB: 683  LB: 640 | UB: 982.2 (909.9, 1058.7)  LB: 920.3 (850.4, 994.5) |
| Reason for Antibiotic Initiation |  |  |  |  |  |  |
| STI Treatment | 42 (57) | 130 | UB: 186.9 (156.2, 222.0)  LB: NA |  | UB: 300  LB: NA | UB: 431.4 (384.0, 483.1)  LB: NA |
| Epidemiologic Treatment for STI Contact | 29 (39) | 64 | UB: 92.0 (70.9, 117.5)  LB: NA |  | UB: 198  LB: NA | UB: 284.7 (246.4, 327.3)  LB: NA |
| Other Health Condition Treatment | 11 (15) | 26 | UB: 37.4 (24.4, 54.8)  LB: NA |  | UB: 185  LB: 142 | UB: 266.0 (229.1, 307.3)  LB: 204.2 (172.0, 240.7) |
| Antibiotic-specific Initiation |  |  |  |  |  |  |
| Doxycycline | 28 (38) | 40 | UB: 58.8 (42.0, 80.1)  LB: 58.8 (42.0, 80.1) |  | UB: 364  LB: 353 | UB: 535.2 (481.6, 593.1)  LB: 518.8 (466.1, 575.8) |
| Azithromycin | 41 (55) | 80 | UB: 117.6 (93.3, 146.4)  LB: 117.6 (93.3, 146.4) |  | UB: 84  LB: 82 | UB: 123.5 (98.5, 152.9)  LB: 120.6 (95.9, 149.7) |
| Ceftriaxone | 41 (55) | 66 | UB: 95.7 (74.0, 121.7)  LB: NA |  | UB: 66  LB: NA | UB: 95.7 (74.0, 121.7)  LB: NA |
| Benzathine-penicillin | 8 (11) | 11 | UB: 15.9 (7.9, 28.4)  LB: NA |  | UB: 11  LB: NA | UB: 15.9 (7.9, 28.4)  LB: NA |
| ^a^ Cumulative number of study participants reporting antibiotic use during follow-up, among 74 total participants  ^b^ Incidence per 100 person-years at risk  ^c^ Upper- and lower-bound estimates for incidence of antibiotic initiation incorporate removal of person-time at risk based on duration ranges for antibiotic regimens outlined in Supplemental Table 1.  ^d^ Upper- and lower-bound estimates for days of antibiotic use are based on ranges for antibiotic regimens outlined in Supplemental Table 1. For estimates without a range of possible regimen durations, the lower bound estimate is listed as NA.  Abbreviations: CI, confidence interval; STI, sexually transmitted infection; UB, upper bound; LB, lower bound; NA, not applicable | | | | | | |

| **Table S2J. Secondary Analysis - Incidence of Antibiotic Initiation & Days of Antibiotic Use Among Study Participants 40+ Years of Age in the ExGen Study, 2016-2018 (N = 52)** | | | | | | |
| --- | --- | --- | --- | --- | --- | --- |
|  |  | Antibiotic Initiation | |  | Days of Antibiotic Use | |
| Outcome | Cumulative Use^a^  N (%) | # of Events | Incidence Rate^b,c^  (95% CI) |  | # of Days | Incidence Rate^b,d^  (95% CI) |
| Any Antibiotic Initiation | 31 (60) | 99 | UB: 202.6 (164.7, 246.7)  LB: NA |  | UB: 775  LB: 728 | UB: 1586.0 (1476.3, 1701.7)  LB: 1489.8 (1383.5, 1602.1) |
| Reason for Antibiotic Initiation |  |  |  |  |  |  |
| STI Treatment | 21 (40) | 56 | UB: 114.6 (86.6, 148.8)  LB: NA |  | UB: 128  LB: NA | UB: 261.9 (218.5, 311.5)  LB: NA |
| Epidemiologic Treatment for STI Contact | 9 (17) | 17 | UB: 34.8 (20.3, 55.7)  LB: NA |  | UB: 68  LB: NA | UB: 139.2 (108.1, 176.4)  LB: NA |
| Other Health Condition Treatment | 13 (25) | 26 | UB: 53.2 (34.8, 78.0)  LB: NA |  | UB: 579  LB: 532 | UB: 1184.9 (1090.3, 1285.4)  LB: 1088.7 (998.1, 1185.3) |
| Antibiotic-specific Initiation |  |  |  |  |  |  |
| Doxycycline | 12 (23) | 19 | UB: 40.2 (24.2, 62.8)  LB: 40.2 (24.2, 62.8) |  | UB: 509  LB: 500 | UB: 1077.3 (985.7, 1175.0)  LB: 1057.7 (967.0, 1154.6) |
| Azithromycin | 23 (44) | 35 | UB: 72.6 (50.6, 101.0)  LB: 72.6 (50.6, 101.0) |  | UB: 51  LB: 47 | UB: 105.8 (78.8, 139.2)  LB: 97.5 (71.7, 129.7) |
| Ceftriaxone | 18 (35) | 23 | UB: 47.3 (30.0, 70.9)  LB: NA |  | UB: 23  LB: NA | UB: 47.3 (30.0, 70.9)  LB: NA |
| Benzathine-penicillin | 4 (8) | 4 | UB: 8.2 (2.2, 21.0)  LB: NA |  | UB: 4  LB: NA | UB: 8.2 (2.2, 21.0)  LB: NA |
| ^a^ Cumulative number of study participants reporting antibiotic use during follow-up, among 52 total participants  ^b^ Incidence per 100 person-years at risk  ^c^ Upper- and lower-bound estimates for incidence of antibiotic initiation incorporate removal of person-time at risk based on duration ranges for antibiotic regimens outlined in Supplemental Table 1.  ^d^ Upper- and lower-bound estimates for days of antibiotic use are based on ranges for antibiotic regimens outlined in Supplemental Table 1. For estimates without a range of possible regimen durations, the lower bound estimate is listed as NA.  Abbreviations: CI, confidence interval; STI, sexually transmitted infection; UB, upper bound; LB, lower bound; NA, not applicable | | | | | | |

| **Table S3. Days of Antibiotic Use for the Treatment of Other Health Conditions Among Study Participants in the ExGen Study, 2016-2018** | | | |
| --- | --- | --- | --- |
| Health Condition | # of Study Participants^a^ | # of Days (% of Total Days for Other Health Conditions)^b^ | |
|  | N (%) | Lower Bound | Upper Bound |
| Acne and Chronic Skin Infections^c^ | 2 (1.4%) | 511 (60.3%) | 511 (54.0%) |
| Acute Skin and Soft Tissue Infections | 8 (5.7%) | 73 (8.6%) | 126 (13.3%) |
| Dental Infections | 2 (1.4%) | 14 (1.7%) | 28 (3.0%) |
| Gastrointestinal Infections | 4 (2.9%) | 16 (1.9%) | 20 (2.1%) |
| Genitourinary Infections | 2 (1.4%) | 23 (2.7%) | 27 (2.9%) |
| Respiratory and ENT (Ear, Nose, Throat) Infections | 11 (7.9%) | 184 (21.7%) | 202 (21.3%) |
| Not Reported | 7 (5.0%) | 27 (3.2%) | 33 (3.5%) |
| Total | 26 (18.6%) | 848 (100%) | 947 (100%) |
| ^a^ Cumulative number of study participants reporting antibiotic use by condition, among 140 total participants. As participants could take antibiotics for multiple conditions, the estimate for ‘Total’ will not equal the sum of all conditions.  ^b^ Upper- and lower-bound estimates for days of antibiotic use are based on ranges for antibiotic regimens outlined in Supplemental Table 1.  ^c^ No range for acne and chronic skin infection regimens, as duration was the time reporting use only. | | | |

| **Table S4. Factors Associated with Antibiotic Initiation for STI Treatment in the ExGen Study, 2016-2018** | | | | | | |
| --- | --- | --- | --- | --- | --- | --- |
| Predictors |  | Univariate Analysis^a^ | |  | Multivariate Analysis^a^ | |
|  |  | IRR (95% CI) | p-value |  | IRR (95% CI) | p-value |
| HIV/PrEP Status |  |  |  |  |  |  |
| PNWH, not on PrEP |  | 1.00 | -- |  | -- | -- |
| PNWH, on PrEP |  | 1.28 (0.63, 2.61) | 0.49 |  | -- | -- |
| PWH |  | 1.23 (0.65, 2.31) | 0.53 |  | -- | -- |
| Age Group |  |  |  |  |  |  |
| 40+ years |  | 1.00 | -- |  | 1.00 | -- |
| 25 – 39 years |  | 1.63 (0.98, 2.70) | 0.06 |  | 1.58 (0.93, 2.69) | 0.09 |
| 18 – 24 years |  | 1.06 (0.48, 2.34) | 0.88 |  | 1.22 (0.47, 3.17) | 0.68 |
| History of chronic medical conditions^b^ |  | 1.02 (0.65, 1.60) | 0.93 |  | -- | -- |
| Reported methamphetamine use^c^ |  | 0.85 (0.53, 1.37) | 0.51 |  | -- | -- |
| Race/ethnicity |  |  |  |  |  |  |
| Non-Hispanic White/Caucasian |  | 1.00 | -- |  | 1.00 | -- |
| Hispanic/Latinx |  | 1.07 (0.59, 1.92) | 0.83 |  | 0.99 (0.52, 1.87) | 0.97 |
| Non-Hispanic Black/African American |  | 0.84 (0.43, 1.66) | 0.62 |  | 1.00 (0.47, 2.13) | 0.99 |
| Another Race/Ethnicity |  | 0.61 (0.30, 1.23) | 0.17 |  | 0.71 (0.35, 1.41) | 0.32 |
| History of bacterial STI diagnosis, <12 months prior to enrollment^d^ |  | 2.24 (1.18, 4.25) | 0.01 |  | 2.18 (1.16, 4.11) | 0.02 |
| ^a^ Implemented negative binomial regression with generalized estimating equations  ^b^ Compared to no history of chronic medical conditions  ^c^ Compared to no reported methamphetamine use  ^d^ Compared to no history of bacterial STI diagnosis, <12 months prior to enrollment  Abbreviations: IRR, incidence rate ratio; CI, confidence interval; PWH, people living with HIV; PNWH, people not living with HIV; PrEP, pre-exposure prophylaxis; STI, sexually transmitted infection | | | | | | |
